# Clinical Characteristics of Cataract and Visual Impairment in Working‐Age Adults: A 9‐Year Retrospective Cohort Study

**DOI:** 10.1155/joph/6062456

**Published:** 2026-10-29

**Authors:** Su Xu, Jingzhi Shao, Jingwen Zhang, Shanshan Du, Kexing Yan, Fengyan Zhang, Yi Mao

**Affiliations:** ^1^ The Division of Ophthalmology and Vision Science Department of Ophthalmology The First Affiliated Hospital of Zhengzhou University Zhengzhou University Zhengzhou China zzu.edu.cn; ^2^ Henan International Joint Research Laboratory for Ocular Immunology and Retinal Injury Repair Henan Province Engineering Research Center of Fundus Disease and Ocular Trauma Prevention and Treatment Henan Children Eye Disease Prevention and Control Engineering Research Center Institute of Ocular Trauma of Zhengzhou University Zhengzhou China

**Keywords:** cataract, epidemiology, visual acuity, visual impairment, working-age adults

## Abstract

**Purpose:**

To characterize cataract‐related visual impairment and clinical features in adults aged 18–50 years and to compare unilateral and bilateral cataract cases.

**Methods:**

This single‐center retrospective cohort study reviewed data from patients aged 18–50 years who were diagnosed with cataract between January 2016 and October 2024. Demographic characteristics, systemic and ocular comorbidities, medication history, lifestyle‐related factors, cataract subtype, and ophthalmic examination findings were collected and compared between unilateral and bilateral cases.

**Results:**

A total of 623 patients were included, comprising 272 patients with unilateral cataracts and 351 with bilateral cataracts. The mean age was 40.73 ± 7.55 years, and 278 patients were female. Frequently observed comorbid conditions included hyperlipidemia, high myopia, prior vitrectomy, prior excimer laser surgery, prior trabeculectomy, and uveitis. Posterior subcapsular cataract was the most common subtype in both unilateral and bilateral cases. Compared with bilateral cataract patients, unilateral cataract patients were diagnosed at a younger age, had worse best‐corrected visual acuity (BCVA) at presentation, and had higher rates of prior vitrectomy and uveitis.

**Conclusions:**

Cataract in working‐age adults was frequently accompanied by metabolic and ocular comorbidities. Unilateral cataracts were characterized by younger age at diagnosis and poorer BCVA at presentation. Prior vitrectomy remained associated with unilateral cataract after multivariable adjustment. These findings may help ophthalmologists and clinicians identify younger cataract patients who require closer monitoring and individualized assessment.

## 1. Introduction

Cataract remains a major cause of visual impairment worldwide and has been most extensively studied in older adults [1–3]. Although advances in cataract care have improved visual outcomes, cataract‐related visual impairment in younger and working‐age adults may affect daily activities, productivity, and quality of life. Compared with the extensive evidence available for age‐related cataract, studies focusing on cataract in younger adults remain relatively limited [4]. In addition, younger cataract patients are more likely to have underlying systemic conditions [5], suggesting that systemic and ocular factors may deserve greater attention in this population. However, the systemic and ocular characteristics, cataract types, and patterns of visual impairment in younger adults remain insufficiently described.

In this study, we aimed to investigate the clinical characteristics of patients diagnosed with cataracts between the ages of 18 and 50 years. We compared demographic characteristics, systemic diseases, ocular history, medication use, lifestyle‐related factors, and ophthalmic examination findings between patients with unilateral and bilateral cataracts. By identifying clinical patterns in this population, our findings may support earlier recognition, ophthalmic risk assessment, and individualized monitoring of working‐age adults with cataract.

## 2. Methods

### 2.1. Patient Inclusion Criteria

This single‐center, 9‐year retrospective observational cohort study included 623 patients aged 18–50 years who were diagnosed with unilateral or bilateral cataracts at a tertiary care center between January 2016 and October 2024. The inclusion criteria were as follows: (1) age between 18 and 50 years; (2) availability of complete medical records; and (3) ability to complete the questionnaire. Patients with traumatic cataract or a documented history of visual impairment since childhood were excluded (Figure 1). Traumatic cataract was excluded because it has a clearly identifiable external cause and differs etiologically from nontraumatic cataract in working‐age adults. Patients with documented visual impairment since childhood were excluded because such cases may include congenital cataract, amblyopia, or other congenital ocular abnormalities that could not be reliably distinguished from acquired cataract in the retrospective records. Eligible patients were required to have complete medical records and available questionnaire data on lifestyle and environmental factors.

**FIGURE 1:**
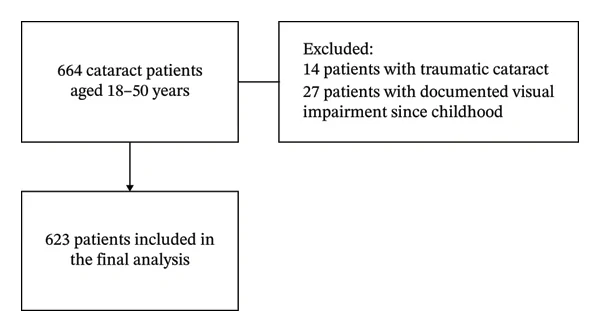
Flowchart of patient selection. Patients with traumatic cataract or a documented history of visual impairment since childhood were excluded.

### 2.2. Ethics Approval

The study was approved by the institutional ethics review board of The First Affiliated Hospital of Zhengzhou University (approval no. 2022‐KY‐0006‐001) and adhered to the Declaration of Helsinki. All participants provided informed consent, and all data were anonymized and de‐identified before analysis.

### 2.3. Clinical Examination and Data Collection

After pharmacologic mydriasis, all participants underwent a comprehensive ophthalmic examination. Cataract diagnosis and subtype classification were determined by an experienced ophthalmologist based on the ophthalmic examination findings documented in the medical records. Cataracts were classified as cortical (CC), nuclear (NC), posterior subcapsular (PSC), hypermature, or mixed cataracts. A standardized quantitative grading of cataract severity was not consistently available in the retrospective medical records; therefore, cataract severity was not included in the between‐group comparisons. Nystagmus and strabismus were documented, and anterior chamber inflammation was assessed. The following ocular parameters were measured: Axial length (AL): Determined using the IOL Master 500 or IOL Master 700. Intraocular pressure (IOP): Measured using a noncontact tonometer. Anterior segment assessment: The zonular integrity, ciliary body, and anterior chamber angle were evaluated using ultrasound biomicroscopy. Anterior segment parameters such as anterior chamber depth (ACD), lens thickness (LT), white‐to‐white (WTW), and dysfunctional lens index (DLI) were measured. Posterior segment assessment: The vitreous, retina, macula, and optic disc were examined using B‐scan ultrasonography, fundus photography, and optical coherence tomography. Demographic data, systemic disease history, medication use, and ophthalmic examination findings were recorded. Additionally, all participants completed a structured questionnaire adapted from a previously published questionnaire on risk factors for early‐onset cataract to assess lifestyle and environmental factors [6] (Figure 2). Although this questionnaire was based on a previously published early‐onset cataract study, it was not a formally validated lifestyle assessment instrument; therefore, lifestyle‐related variables were analyzed as exploratory factors and interpreted with caution.

**FIGURE 2:**
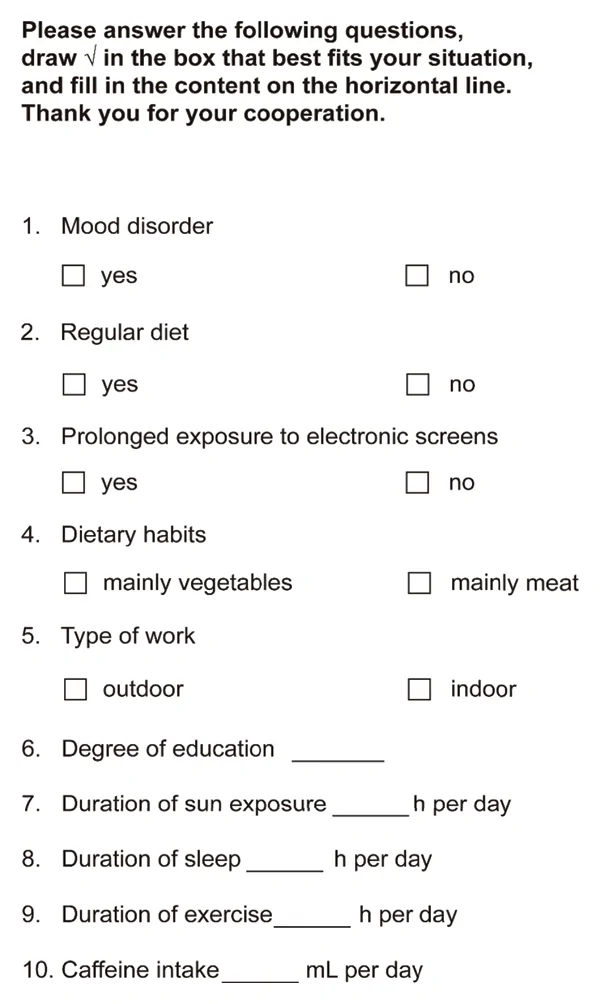
Structured questionnaire on lifestyle and environmental factors. The questionnaire was adapted from a previously published questionnaire on risk factors for early‐onset cataract.

Best‐corrected visual acuity (BCVA) at presentation was recorded and converted to logarithm of the minimum angle of resolution (logMAR) values for statistical analysis. Decimal visual acuity was converted using the formula logMAR = −log10(decimal visual acuity). For very low visual acuity, counting fingers (CF), hand motion (HM), light perception (LP), and no light perception (NLP) were assigned logMAR values of 1.9, 2.3, 2.7, and 3.0, respectively, according to previously published methods [7]. For patients with bilateral cataract, one eye was randomly selected for eye‐level analysis. All eye‐specific information, including cataract subtype, BCVA at presentation, ocular comorbidities, previous ocular procedures, and ophthalmic examination parameters, corresponded to the selected eye. Systemic diseases, medication history, and lifestyle‐related factors were recorded at the patient level and were not affected by eye selection.

### 2.4. Statistical Analysis

The normality of continuous variables was assessed using the Shapiro–Wilk test together with visual inspection of Q‐Q plots. Normally distributed continuous variables were expressed as mean ± standard deviation and compared using independent samples *t* tests. Welch’s *t* test was used when the assumption of equal variances was not met. Non‐normally distributed continuous variables were expressed as median and interquartile range and compared using the Mann–Whitney *U* test. Categorical variables were presented as number and percentage (*n* [%]) and compared using the chi‐square test or Fisher’s exact test, as appropriate. Multivariable binary logistic regression analysis was performed to further evaluate factors associated with cataract laterality and to reduce the influence of potential confounding in the comparison between unilateral and bilateral cataract. Cataract laterality was entered as the dependent variable (bilateral = 0 and unilateral = 1), and sex, age, prior vitrectomy, uveitis, BCVA, and high myopia were included in the model. These variables were selected based on clinical relevance and the results of between‐group comparisons. Model calibration was assessed using the Hosmer–Lemeshow goodness‐of‐fit test. Adjusted odds ratios (ORs) and 95% confidence intervals (CIs) were reported. A sensitivity analysis was performed after excluding patients with prior vitrectomy and/or uveitis to determine whether the main differences between unilateral and bilateral cataract were influenced by these eye‐specific conditions. All analyses were considered exploratory because of the retrospective observational design. A two‐sided *p* < 0.05 was considered statistically significant.

## 3. Results

### 3.1. Patient Population Characteristics

A total of 623 patients were included in the study, with a mean age of 40.73 ± 7.55 years. The male‐to‐female ratio was 1.24:1 (345 males, 278 females). Among these patients, 272 (43.7%) had unilateral cataracts, while 351 (56.3%) had bilateral cataracts. The demographic and clinical characteristics of the study population are summarized in Table 1.

**TABLE 1:** General characteristics of cataract patients aged 18–50 years.

Parameter	Mean ± SD	Min	Max
Age (years)	40.73 ± 7.55	18.00	50.00
BMI	24.58 ± 3.74	16.79	40.00
AL (mm)	25.30 ± 2.84	20.14	36.00
ACD (mm)	3.35 ± 0.51	1.62	5.32
LT (mm)	4.02 ± 0.78	1.00	7.00
WTW (mm)	11.91 ± 0.51	10.00	12.10
DLI	2.15 ± 2.08	0.36	10.00
Sun exposure (h/day)	1.33 ± 1.69	0.00	10.00
Sleep duration (h/day)	7.12 ± 0.79	4.50	10.00
Exercise duration (h/day)	0.42 ± 0.45	0.00	4.00
Caffeine intake (mL/day)	91.96 ± 146.70	0.00	1000.00

### 3.2. Comorbid Conditions

The distribution of systemic and ocular comorbid conditions among the study population is presented in Table 2. Hyperlipidemia, defined as hypertriglyceridemia and/or hypercholesterolemia, was the most frequently observed systemic comorbidity. In addition to common metabolic disorders such as hypertension and diabetes, atopic conditions were identified in 13.0% of patients, with drug allergy being the most prevalent manifestation.

**TABLE 2:** Comorbidities among cataract patients aged 18–50 years.

Comorbidities	*n* (%)
*Systemic*	
Hypertriglyceridemia	246 (39.5)
Hypercholesterolemia	90 (14.4)
Hypertension	89 (14.3)
Atopic conditions (allergy, asthma, etc.)	81 (13.0)
Diabetes	62 (10.0)

*Ocular*	
High myopia	194 (31.1)
Prior vitrectomy	69 (11.1)
Prior excimer laser therapy	35 (5.6)
Uveitis	31 (5.0)
Prior trabeculectomy	28 (4.5)

*Note:* This table presents the five most prevalent systemic and the five most prevalent ocular comorbidities.

Based on patient history and clinical examinations, the most common ocular comorbid condition, aside from cataracts, was high myopia, followed by a history of vitrectomy. Additionally, a notable proportion of patients had undergone previous excimer laser therapy, experienced uveitis, or had a history of trabeculectomy (Table 2).

### 3.3. Comparison of Clinical Characteristics Between Unilateral and Bilateral Cataract Patients

To further characterize cataract in working‐age adults, we compared the clinical characteristics of patients with unilateral and bilateral cataracts. PSC was the most prevalent subtype in both groups (Figure 3). Patients with unilateral cataracts were more likely to have a history of vitrectomy than those with bilateral cataracts (18.0% vs. 5.7%, *p* < 0.001) and uveitis (6.9% vs. 3.4%, *p* = 0.042). They were also diagnosed at a younger mean age than patients with bilateral cataracts (39.75 vs. 41.49 years, *p* = 0.005). Notably, BCVA at presentation was worse in unilateral cases than in bilateral cases (1.24 vs. 1.05 logMAR, *p* < 0.001), indicating greater visual impairment at presentation. Bilateral cataract patients had higher rates of selected ocular and systemic comorbidities, including pterygium, epiretinal membrane, retinitis pigmentosa, urinary system diseases, and corticosteroid or immunosuppressant use (Figure 4). No significant differences were observed between the two groups in anterior segment parameters or lifestyle factors. Multivariable logistic regression analysis showed that male sex (adjusted odds ratio [aOR] = 1.469, 95% CI: 1.050–2.055, *p* = 0.025), younger age (aOR per year = 0.976, 95% CI: 0.954–0.998, *p* = 0.033), prior vitrectomy (aOR = 3.504, 95% CI: 1.979–6.204, *p* < 0.001), and poorer BCVA (aOR per 1‐logMAR increase = 1.370, 95% CI: 1.060–1.772, *p* = 0.016) were associated with unilateral cataract. High myopia was associated with lower odds of unilateral cataract (aOR = 0.655, 95% CI: 0.454–0.945, *p* = 0.024), whereas uveitis was not significantly associated with cataract laterality after adjustment (aOR = 1.743, 95% CI: 0.790–3.845, *p* = 0.169) (Table 3). The Hosmer–Lemeshow goodness‐of‐fit test showed no evidence of poor model fit (*p* = 0.469). In the sensitivity analysis excluding patients with prior vitrectomy and/or uveitis, patients with unilateral cataract remained younger than those with bilateral cataract (40.18 ± 7.70 vs. 41.83 ± 6.83 years; mean difference, 1.655 years; 95% CI: 0.358–2.952, *p* = 0.013) and had poorer BCVA (1.14 ± 0.69 vs. 1.01 ± 0.60 logMAR; mean difference, 0.125 logMAR; 95% CI: 0.010–0.240, *p* = 0.034).

**FIGURE 3:**
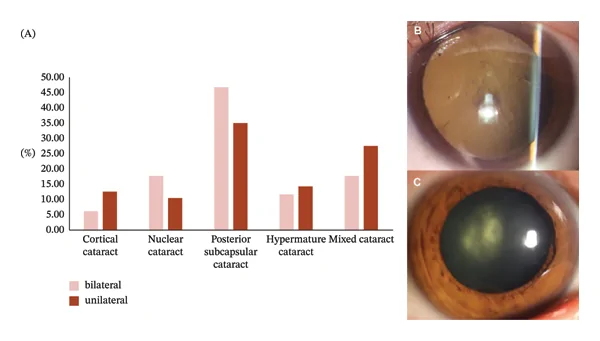
Comparison of cataract subtypes in patients with unilateral and bilateral cataracts. (A) Distribution of cataract subtypes, with posterior subcapsular cataract being the most common subtype. (B, C) Typical examples of posterior subcapsular cataracts.

**FIGURE 4:**
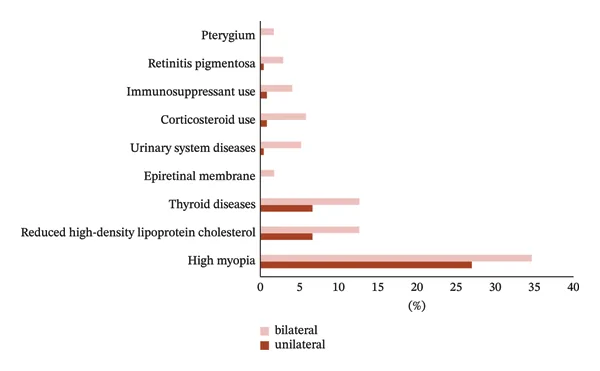
Comparison of comorbidities and medication use between patients with unilateral and bilateral cataracts. Only variables with statistically significant between‐group differences are shown.

**TABLE 3:** Multivariable logistic regression analysis of factors associated with unilateral cataract.

Variable	Adjusted OR	95% CI	*p* value
Male sex	1.469	1.050–2.055	0.025
Age, per year	0.976	0.954–0.998	0.033
Prior vitrectomy	3.504	1.979–6.204	< 0.001
Uveitis	1.743	0.790–3.845	0.169
BCVA, per 1‐logMAR increase	1.370	1.060–1.772	0.016
High myopia	0.655	0.454–0.945	0.024

*Note:* Bilateral cataract was used as the reference category for the dependent variable. Female sex, no prior vitrectomy, no uveitis, and no high myopia were used as the reference categories for categorical variables.

Abbreviations: aOR, adjusted odds ratio; BCVA, best‐corrected visual acuity; CI, confidence interval.

## 4. Discussion

This study characterized cataract‐related visual impairment and clinical features in adults aged 18–50 years and identified differences between unilateral and bilateral cases to improve the understanding of this population. Anterior segment analysis revealed that younger cataract patients had a deeper ACD than the normal reference values. Given that previous studies have established an association between aging and a progressive decrease in ACD [8], our findings are plausible, as the study population was relatively young. Additionally, the DLI, a novel parameter reflecting lens function, was markedly impaired (mean DLI: 2.15), aligning with the pathophysiological changes characteristic of cataract development [9]. However, because this study did not include an older age‐related cataract group, these comparisons were based on previous reports rather than direct comparisons within this study.

Metabolic abnormalities were frequently observed in this study, with hyperlipidemia affecting more than half of patients and a relatively high mean body mass index (BMI) observed in this working‐age cohort. Prior studies have suggested that hyperlipidemia and low HDL cholesterol levels may accelerate cataract progression in diabetic animal models [10], while epidemiological data, including the Framingham study, have identified hypertriglyceridemia as a risk factor for PSC, particularly in males [11]. A cross‐sectional case analysis involving 219 cataract patients showed that elevated serum triglyceride (TG) levels were an independent risk factor for cataracts [12]. Additionally, studies based on the GBD study have shown that the prevalence of cataracts, especially PSC, has been linked to a high BMI [13]. These results support the inclusion of metabolic history in clinical assessment and risk recognition for younger adults with cataract.

Beyond metabolic factors, systemic conditions such as hypertension [14], diabetes [14], and atopic diseases [6] were also observed in this cohort. These conditions have been linked to cataract through inflammatory, osmotic, or immune‐mediated pathways, though causal relationships cannot be inferred from this study. Previous studies have implicated hypertension in cataracts through inflammatory pathways (including interleukin‐6 (IL‐6) and tumor necrosis factor‐alpha (TNF‐α) regulation), structural alterations in the lens capsule, and potential genetic predisposition, with these mechanisms acting independently of hyperglycemia and dyslipidemia [15]. Diabetes promotes cataract formation through aldose reductase‐mediated osmotic stress, leading to lens fiber damage and subsequent opacification [16, 17]. In addition, atopic conditions such as asthma and allergies may contribute to cataract development through autoantibody‐mediated damage to lens epithelial cells (LECs) or eosinophil‐induced inflammation [18–20].

High myopia [21], previous vitrectomy [22], prior excimer laser surgery [23], uveitis [24], and trabeculectomy [25] were common ocular histories in this cohort and may be clinically relevant when evaluating younger cataract patients. High myopia may accelerate lens opacification through persistent microtrauma, vitreous liquefaction, and zonular instability, ultimately compromising lens transparency [21]. Moreover, a history of intraocular surgery has been shown to increase the risk of cataract progression, likely due to surgical trauma and secondary inflammatory responses [26–29]. These findings highlight the need for close monitoring of high‐risk individuals and emphasize the importance of early screening and intervention in young patients to mitigate cataract development.

In the comparison between unilateral and bilateral cataracts, unilateral cases showed worse BCVA at presentation and higher rates of prior vitrectomy and uveitis, whereas bilateral cases had higher rates of selected ocular and systemic comorbidities, including pterygium, epiretinal membrane, retinitis pigmentosa, urinary system diseases, and corticosteroid or immunosuppressant use. These findings suggest that unilateral and bilateral cataracts in working‐age adults may differ in several clinical characteristics. Local ocular history, particularly prior vitrectomy and ocular inflammation, may be more relevant in unilateral cases. However, previous vitrectomy and uveitis are eye‐specific factors that may themselves contribute to cataract development. Therefore, the observed differences should not be interpreted as an independent effect of cataract laterality, and differences in local ocular history should be considered when interpreting these results. Importantly, after excluding patients with prior vitrectomy and/or uveitis, unilateral cataract patients remained significantly younger and had poorer BCVA than bilateral cataract patients. This sensitivity analysis suggests that these two differences were not entirely explained by prior vitrectomy or uveitis. After adjustment for potential confounding factors, younger age, poorer BCVA, prior vitrectomy, and male sex remained associated with unilateral cataract, whereas high myopia was associated with lower odds of unilateral cataract. Although uveitis was more frequent in unilateral cases in the unadjusted comparison, this association was no longer statistically significant after multivariable adjustment.

Beyond the poorer BCVA at presentation, this study also found that patients with unilateral cataracts were diagnosed at a younger age. Earlier diagnosis in unilateral cases may reflect a greater contribution of local ocular factors rather than systemic aging alone. The younger age at diagnosis in unilateral cataract patients underscores the need for enhanced early screening and targeted preventive measures to mitigate visual impairment. Additionally, among adults aged 18–50 years, PSC, which has a significant impact on vision and tends to progress rapidly, was identified as the most common subtype, regardless of whether one or both eyes were affected. However, because detailed cataract subtype grading was not uniformly documented in the retrospective records and mixed cataracts were common, further subtype‐specific association analyses were not considered sufficiently reliable. This finding highlights the importance of early detection and intervention in this population to prevent further visual deterioration.

Several clinical differences were observed between unilateral and bilateral cataract cases. The younger age at diagnosis and poorer BCVA at presentation in patients with unilateral cataracts indicate a potential need for more proactive clinical assessment and monitoring. A history of vitrectomy and ocular inflammation should be carefully considered during clinical assessment and monitoring of working‐age cataract patients.

Several systemic and ocular factors identified in this study have also been reported in previous studies. The novelty and strength of this study lie in the comprehensive characterization of cataract in working‐age adults using a relatively large 9‐year cohort of 623 patients aged 18–50 years. Unlike many previous studies that focused on isolated risk factors or age‐related cataract, our study jointly evaluated systemic diseases, ocular history, medication use, lifestyle‐related factors, cataract subtype, and ophthalmic findings, and further compared unilateral and bilateral cataract cases. Therefore, the present findings mainly extend previous observations in working‐age adults rather than identify entirely new cataract risk factors. This study has several limitations. First, it was a single‐center, hospital‐based retrospective study, which may limit generalizability. Second, the absence of a noncataract control group prevents estimation of independent risk factors. Third, lifestyle‐related variables were collected using a structured questionnaire adapted from a previous study on early‐onset cataract. However, this questionnaire was not a formally validated lifestyle assessment instrument, and the self‐reported data may be subject to recall bias. Therefore, lifestyle‐related findings should be interpreted as exploratory. Future prospective studies should adopt established and validated lifestyle instruments to achieve more quantitative and comparable assessment of lifestyle‐related exposures. In addition, detailed cataract subtype grading was not uniformly documented in the retrospective records, and mixed cataracts were common; therefore, reliable subtype‐specific association analyses were not feasible. Serial standardized assessments of lens opacity were also unavailable, preventing evaluation of cataract progression rate. Finally, the exploratory analyses did not establish causal relationships. Future multicenter prospective studies incorporating standardized visual function measures and longitudinal follow‐up are warranted to clarify the clinical course and risk profile of cataract in working‐age adults. Such research could also enhance public awareness and understanding of cataracts in adults aged 18–50 years, ultimately contributing to the development of more effective clinical management strategies.

## 5. Conclusions

In adults aged 18–50 years, cataract was frequently accompanied by metabolic and ocular comorbidities. Unilateral cataracts were characterized by younger age at diagnosis and worse BCVA at presentation. These findings may help ophthalmologists and clinicians recognize working‐age patients who need closer monitoring and individualized assessment.

## Author Contributions

Su Xu: conceptualization, data curation, methodology, and writing–original draft. Jingzhi Shao: conceptualization and writing–review and editing. Jingwen Zhang and Kexing Yan: data curation. Shanshan Du: writing–review and editing. Fengyan Zhang: project administration, funding acquisition and writing–review and editing. Yi Mao: project administration, funding acquisition, and writing–review and editing.

## Funding

This study was supported by the China Postdoctoral Science Foundation (No. 2023M733238), the Joint Construction Project of Henan Provincial Medical Science and Technology Research Program (No. LHGJ2023170), Natural Science Foundation of Henan Province (No. 252300421593), and the Doctor Project in Central Plains (11679), Henan province, China.

## Disclosure

All authors approved the final version of the manuscript. The funding agencies had no role in study design, data collection and analysis, decision to publish, or preparation of the manuscript.

## Conflicts of Interest

The authors declare no conflicts of interest.

## Data Availability

The datasets generated and analyzed during the current study are not publicly available because of patient privacy and ethical restrictions. De‐identified data may be available from the corresponding author on reasonable request and with appropriate ethical approval.

## References

[1] Naidoo K. , Kempen J. H. , Gichuhi S. et al., Prevalence and Causes of Vision Loss in Sub-Saharan Africa in 2015: Magnitude, Temporal Trends and Projections, British Journal of Ophthalmology. (2020) 104, no. 12, 1658–1668, 10.1136/bjophthalmol-2019-315217. 32229517

[2] Lee C. M. and Afshari N. A. , The Global State of Cataract Blindness, Current Opinion in Ophthalmology. (2017) 28, no. 1, 98–103, 10.1097/ICU.0000000000000340. 27820750

[3] Cicinelli M. V. , Buchan J. C. , Nicholson M. , Varadaraj V. , and Khanna R. C. , Cataracts, Lancet. (2023) 401, no. 10374, 377–389, 10.1016/S0140-6736(22)01839-6. 36565712

[4] Hashemi H. , Pakzad R. , Aghamirsalim M. et al., Age- and Sex-Standardized Prevalence of Age-Related Cataract and Its Determinants; Tehran Geriatric Eye Study (TGES), Ophthalmic Epidemiology. (2022) 29, no. 5, 554–565, 10.1080/09286586.2021.1976804. 34592897

[5] Bernth-Petersen P. and Bach E. , Epidemiologic Aspects of Cataract Surgery. III: Frequencies of Diabetes and Glaucoma in a Cataract Population, Acta Ophthalmologica. (1983) 61, no. 3, 406–416, 10.1111/j.1755-3768.1983.tb01439.x. 6624407

[6] Praveen M. R. , Shah G. D. , Vasavada A. R. , Mehta P. G. , Gilbert C. , and Bhagat G. , A Study to Explore the Risk Factors for the Early Onset of Cataract in India, Eye. (2010) 24, no. 4, 686–694, 10.1038/eye.2009.137. 19521430

[7] Sahanne S. , Tuuminen R. , Haukka J. , and Loukovaara S. , A Retrospective Study Comparing Outcomes of Primary Rhegmatogenous Retinal Detachment Repair by Scleral Buckling and Pars Plana Vitrectomy in Finland, Clinical Ophthalmology. (2017) 11, 503–509, 10.2147/OPTH.S128746. PMC5354527 28331284

[8] Isidore M. , Hoa D. , Verhaeghe F. et al., Discordance Between Anterior Segment Anatomy and Axial Length in Cataract Surgery Candidates in France, Ophthalmic Research. (2023) 66, no. 1, 474–480, 10.1159/000526281. 36455535

[9] Kaweri L. , Wavikar C. , James E. , Pandit P. , and Bhuta N. , Review of Current Status of Refractive Lens Exchange and Role of Dysfunctional Lens Index as Its New Indication, Indian Journal of Ophthalmology. (2020) 68, no. 12, 2797–2803, 10.4103/ijo.IJO_2259_20. PMC7856935 33229654

[10] Tsutsumi K. , Inoue Y. , and Yoshida C. , Acceleration of Development of Diabetic Cataract by Hyperlipidemia and Low High-Density Lipoprotein in Rats, Biological and Pharmaceutical Bulletin. (1999) 22, no. 1, 37–41, 10.1248/bpb.22.37. 9989659

[11] Hiller R. , Sperduto R. D. , Reed G. F. , D’Agostino R. B. , and Wilson P. W. F. , Serum Lipids and Age-Related Lens Opacities: A Longitudinal Investigation: The Framingham Studies, Ophthalmology. (2003) 110, no. 3, 578–583, 10.1016/S0161-6420(02)01762-1. 12623825

[12] Li S. , Li D. , Zhang Y. , Teng J. , Shao M. , and Cao W. , Association Between Serum Lipids Concentration and Patients With Age-Related Cataract in China: A Cross-Sectional, Case-Control Study, British Medical Journal Open. (2018) 8, no. 4, 10.1136/bmjopen-2018-021496. PMC5892756 29626052

[13] Wu T. H. , Jiang B. , Liu W. M. , Li J. Q. , Song Z. Y. , and Lu P. R. , Time Trends and Gender Disparities of Chinese Cataract Burden and Their Predictions, International Journal of Ophthalmology. (2023) 16, no. 9, 1527–1534, 10.18240/ijo.2023.09.21. PMC10475628 37724286

[14] Sarkar D. , Sharma R. , Singh P. et al., Age-Related Cataract-Prevalence, Epidemiological Pattern and Emerging Risk Factors in a Cross-Sectional Study From Central India, Indian Journal of Ophthalmology. (2023) 71, no. 5, 1905–1912, 10.4103/ijo.IJO_2020_22. PMC10391463 37203054

[15] Yu X. , Lyu D. , Dong X. , He J. , and Yao K. , Hypertension and Risk of Cataract: A Meta-Analysis, Public Library of Science One. (2014) 9, no. 12, 10.1371/journal.pone.0114012. PMC4256215 25474403

[16] Cogan D. G. , Kinoshita J. H. , Kador P. F. et al., NIH Conference. Aldose Reductase and Complications of Diabetes, Annals of Internal Medicine. (1984) 101, no. 1, 82–91, 10.7326/0003-4819-101-1-82. 6203454

[17] Kinoshita J. H. , Fukushi S. , Kador P. , and Merola L. O. , Aldose Reductase in Diabetic Complications of the Eye, Metabolism. (1979) 28, no. 4 Suppl 1, 462–469, 10.1016/0026-0495(79)90057-x. 45423

[18] Yamamoto N. , Hiramatsu N. , Isogai S. , Kondo M. , Imaizumi K. , and Horiguchi M. , Mechanism of Atopic Cataract Caused by Eosinophil Granule Major Basic Protein, Medical Molecular Morphology. (2020) 53, no. 2, 94–103, 10.1007/s00795-019-00234-5. 31595373

[19] Cordes F. C. and Cordero-Moreno R. , Atopic Cataracts; Report of Four Cases, American Journal of Ophthalmology. (1946) 29, 402–407. 21019946

[20] Hori K. , Matsuda A. , Ebihara N. et al., Involvement of Plasminogen Activator Inhibitor-1 in the Pathogenesis of Atopic Cataracts, Investigative Ophthalmology & Visual Science. (2012) 53, no. 4, 1846–1851, 10.1167/iovs.11-8380. 22410565

[21] Yu H. J. , Kuo M. T. , and Wu P. C. , Clinical Characteristics of Presenile Cataract: Change over 10 Years in Southern Taiwan, Biomed Research International. (2021) 2021, no. 1, 10.1155/2021/9385293. PMC8016560 33834076

[22] Blodi B. A. and Paluska S. A. , Cataract After Vitrectomy in Young Patients, Ophthalmology. (1997) 104, no. 7, 1092–1095, 10.1016/S0161-6420(97)30180-8. 9224458

[23] Ortiz-Morales G. , Ramos-Davila E. M. , Elizondo-Fernández B. , Arreola-Martinez J. , Garcia J. N. , and Valdez-Garcia J. E. , LASIK is Associated With Early Cataract Surgery in Healthy Patients, International Ophthalmology. (2024) 44, no. 1, 10.1007/s10792-024-03060-6. 38431898

[24] Papaliodis G. N. , Rosner B. A. , Dreger K. A. et al., Incidence of and Risk Factors for Cataract in Anterior Uveitis, American Journal of Ophthalmology. (2023) 254, 221–232, 10.1016/j.ajo.2023.06.021. PMC10528977 37414328

[25] Adelman R. A. , Brauner S. C. , Afshari N. A. , and Grosskreutz C. L. , Cataract Formation After Initial Trabeculectomy in Young Patients, Ophthalmology. (2003) 110, no. 3, 625–629, 10.1016/S0161-6420(02)01769-4. 12623833

[26] Holekamp N. M. , Shui Y. B. , and Beebe D. C. , Vitrectomy Surgery Increases Oxygen Exposure to the Lens: A Possible Mechanism for Nuclear Cataract Formation, American Journal of Ophthalmology. (2005) 139, no. 2, 302–310, 10.1016/j.ajo.2004.09.046. 15733992

[27] D’Ermo F. , Bonomi L. , and Doro D. , A Critical Analysis of the Long-Term Results of Trabeculectomy, American Journal of Ophthalmology. (1979) 88, no. 5, 829–835, 10.1016/0002-9394(79)90559-2. 507161

[28] Watson P. G. and Grierson I. , The Place of Trabeculectomy in the Treatment of Glaucoma, Ophthalmology. (1981) 88, no. 3, 175–196, 10.1016/s0161-6420(81)35051-9. 7231906

[29] Müller-Stolzenburg N. W. , Müller G. J. , Buchwald H. J. , and Schründer S. , UV Exposure of the Lens During 193-mm Excimer Laser Corneal Surgery, Archives of Ophthalmology. (1990) 108, no. 7, 915–916, 10.1001/archopht.1990.01070090017005. 2369344

